# Bilateral cleft lip repair by new trending method: a case report

**DOI:** 10.1186/s40902-022-00367-1

**Published:** 2022-12-14

**Authors:** Ji-Yeon Kang

**Affiliations:** Department of Oral and Maxillofacial Surgery, College of Medicine and Medical Research Institute, Chungnam National University, 282 Munhwa-Ro, Jung-Gu Daejeon, Daejeon, 35015 Korea

**Keywords:** Bilateral cleft lip, Cheiloplasty, Cleft lip repair

## Abstract

**Background:**

Bilateral cleft lip repair is one of the most difficult surgeries, and many techniques have been modified and developed to improve surgical outcomes. The current trend is toward preserving tissue as much as possible. When the reconstruction is based on the shape of the patient’s own tissue, the most natural appearance is produced, and the relaxed remaining tissue can be benefitted from reducing tension and minimizing scarring.

Case presentation.

In the conventional surgical method, the rest of the prolabium is sacrificed, except for the tissue used to make the philtrum. We used all tissues for surgery and did not discard any. The tubercle of the median vermilion was used in its original form.

**Conclusions:**

It is fundamental to restore function in cleft lip surgery. Both patients and surgeons have a desire for esthetic outcomes that go beyond function. In addition, the measure of the success of the surgery is the postoperative resemblance to normal midfacial features. Unlike the conventional method of making tubercles by collecting lateral vermilion flaps, we preserved the tissue of the prolabium. Rather than using an artificial tubercle, we were able to create a more natural shape of the upper lip using the patient’s own anatomical structure. In addition, the remaining tissues of the discarded prolabium were used to make the oral mucosa, which may help to reduce tension compared to the conventional method. The modified repair method is expected to gradually become the mainstream method owing to its superior esthetic outcome and less surgical difficulty compared with traditional methods.

## Background

The goal of cleft lip surgery is to create a normal anatomical structure by suturing the separated upper labial soft tissue. It is critical to reposition the orbicularis oris muscle that has been oriented along the cleft and gain function by achieving muscle continuity. The nasolabial units, which include the columella, philtrum, and Cupid’s bow, as well as the nose, which includes the nasal alar and nasal tip, should be symmetrical. Compared to the lower lip, the upper lip should have an appropriate length, width, and protrusion, and the detailed structure, such as the shape of the philtral ridge, the position of Cupid’s bow peak, and the protrusion of the upper labial tubercle, should also be natural [1–4]. However, obtaining satisfactory surgical results is difficult, particularly in bilateral cleft lip, where various defect types, such as symmetric complete, incomplete, lesser form, and asymmetric, are mixed and asymmetry is often observed before surgery. A lack of tissue or a protruding anterior premaxilla causes tension in the soft tissues and is often accompanied by a cleft palate, which poses a difficulty for the doctor. In addition, a four-dimensional problem approach should be used, considering not only the three-dimensional structure but also the growth of patients undergoing surgery in infancy [5]. These concerns led to the development of principles and techniques through the efforts of scholars. The principle of Mulliken, which is considered the basic principle, is as follows [3]: (1) maintain symmetry, (2) secure primary muscle union, (3) select the proper prolabial size and configuration, (4) create the median tubercle and mucocutaneous ridge from lateral lip tissue, and (5) construct the nasal tip and columella by anatomically positioning the alar cartilages. He also constantly improved his technique and proposed a method for making a philtral flap in the prolabium and making the rest of the structure with lateral lip flaps [6]. This surgical method has been accepted and inspired by many surgeons, and most of the bilateral cleft lip surgeries performed today follow Mulliken’s method with some modifications. However, many patients undergo several repairing surgeries as they become adults. Regarding the lips, the causes of lip deformity include scars, incongruity of lip length, deformation of the philtrum and orbicularis oris muscle, and vermilion deformity. In particular, the median tubercle was not well defined, absent, and often accompanied by whistle-tip deformity due to inadequate bulk provided by the lateral segment vermilion [7]. In the present case, the upper labial median structures, including the Cupid’s bow and tubercle, were taken from the prolabium; additionally, in contrast to the existing method, the remaining prolabial tissue was also used to reduce tension without sacrificing any tissue, resulting in good outcomes. This method contradicts previous concepts, but it is also suggested in other reports. We believe that the paradigm can be gradually changed.

## Case presentation

**Fig. 1:**
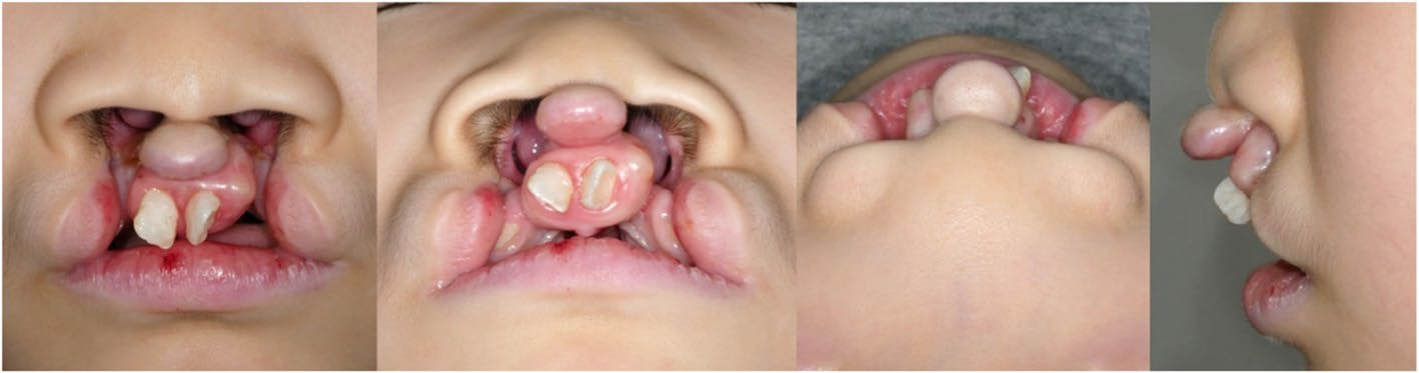
Preoperative photographs

Prior to his initial visit, an 8-year-old child with a bilateral complete cleft lip had received no treatment after birth (Fig. 1). He was found to have an asymmetric bilateral complete cleft palate, nasal deformity, and anterior premaxilla protrusion. However, because cleft palate surgery was not performed, maxillary hypoplasia was not observed.

### Design

**Fig. 2:**
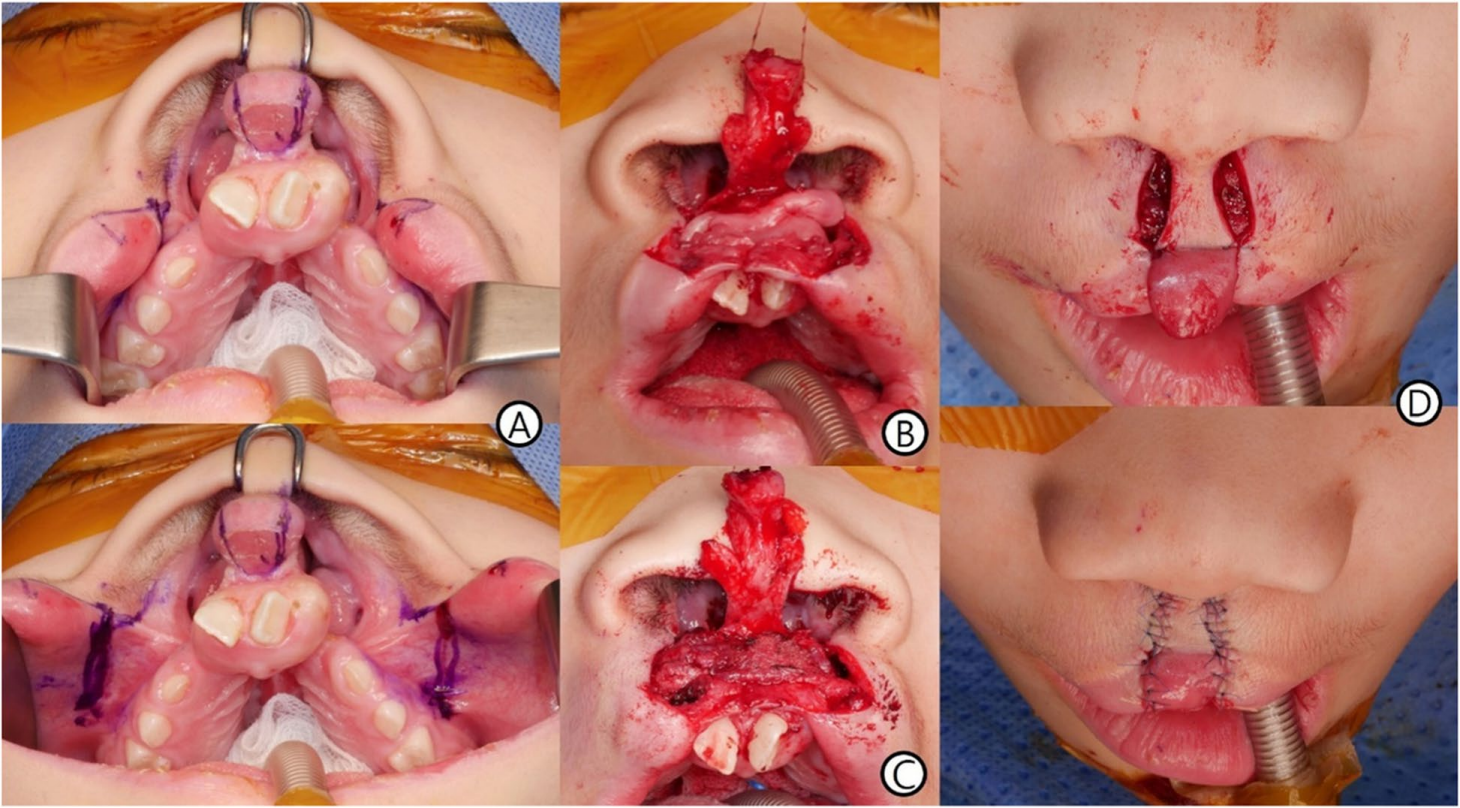
Operative findings. **A** Operative design. **B** Sutured state of the bilateral lateral vermilion flaps in the middle of the lip. **C** Muscle repair. **D** Skin repair

The midpoint of the vermilion and the bilateral Cupid’s bow peaks in the prolabium were marked. We then marked the medial alar border of both nostrils, which was intended to be the upper margin of the philtrum, and created the prolabial flap by connecting the border with the previously marked Cupid’s bow peaks while considering the shape of the philtrum, with the width widened toward the lower part but not completely (i.e., trapezoidal square shape). This line passed through the vermilion and oral mucosa. Furthermore, it extended to the hinge formed by the prolabium and premaxilla and was connected along the hinge. We drew a curved line along the vermilion margin on both sides of the prolabial flap to form a triangular flap. The lateral lip point was marked on the area where the curved line became a straight line on the lateral vermilion and where the white roll disappeared. The length of the lateral lip flap was determined according to the prolabial flap. The line extended into the nose along cleft margins to form a nasal mucosal flap that was intended to form the nasal floor. A lateral vermilion flap was formed by making vertical incisions at the upper and lower edges of the lateral lip flap. The incision on the upper edge extended to the labial sulcus, and the lower edge extended to the red vermilion (Fig. 2).

### Flap elevation

We lifted the prolabial flap and the bilateral triangular flap, including the central vermilion, leaving the incision at the end point where the two flaps separated. The vermilion on both sides, except the raised flaps, was dissected to the premaxilla hinge to form medial vermilion flaps for the oral lining. After elevating the lateral vermilion flap from the lateral lip, we detached the abnormal insertion of the orbicularis oris muscle and dissected it to enable sufficient mobility. Alar cartilage detachments were performed through flap incisions.

### Oral lining and orbicularis oris muscle repair

The lateral vermilion flap was unfolded medially to meet the medial vermilion flap, and the two flaps were sutured together to form the oral lining. The dissected orbicularis oris muscle was sutured end-to-end from the lowest margin to the uppermost margin to achieve continuity.

### Nasal lining and skin repair

The prolabial flap was placed on the orbicularis oris muscle restoring continuity and sutured to the lateral lip flaps to repair the skin. The triangular flaps can be used to lengthen the columella; however, the comumella was already sufficient in length. In addition, the base of the triangular flap was trimmed and sutured to the base of the columellar side.

## Conclusions

Bilateral cleft lip repair has been continuously improved in order to achieve good results. Previously, a two-stage operation was used to be performed on the severe side, considering the blood circulation of the prolabium. However, since the second half of the twentieth century, most clefts have been reconstructed with a single operation on both sides [8]. The surgical scope has also evolved from skin-based closure surgery to a three-dimensional concept that separates muscle and nasal cartilage. Concerns have been raised about not only improving the overall esthetics of the nasolabial complex but also minimizing secondary deformities.

**Fig. 3:**
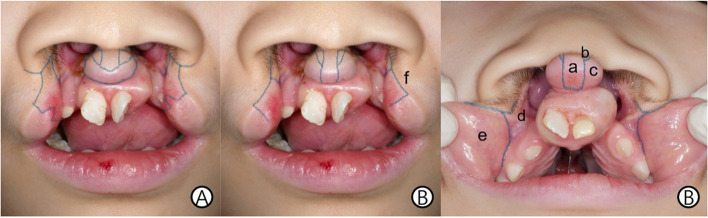
Operative design. **A** Conventional, modified Mulliken method. **B** Modified design containing medial vermilion in the philtral flap. (a) Prolabial flap, (b) triangular flap, (c) medial vermilion flap, (d) nasal mucosal flap, (e) lateral vermilion flap, and (f) lateral lip flap

In accordance with several previous techniques of scholars, such as Veau, Bauer–Trusler–Tondra, Millard, Wynn, Manchester, Mulliken, and Noordhoff, the current surgical methods are based on the straight-line methods such as Millard, Mulliken [6], and Fisher [9] based on the techniques by Mulliken or Chen and Noordhoff [10], rather than the triangular flap or the square flap which requires complicated construction. Although each surgeon’s technique differs slightly, the philtrum flap is obtained from the prolabium. When the lateral lip flap is placed, forked flaps are formed on both sides to increase the columella length, or de-epithelialized strips are formed to raise the philtral margin. The rest of the prolabium is discarded. The lateral vermilion flap is used to make the medial tubercle. Additionally, oral mucosa is created with a lateral lip advancement flap through the vestibular incision and sufficient dissection extending from the labial sulcus. The lateral lip flap may be curved to extend to the lower lateral side of the nasal wing, or the lower lateral cartilage may be accessed via a nostril rim incision (Fig. 3). However, most surgeons access it through a flap incision and do not require additional intranasal incisions to dissect the alar cartilage. In addition, interdormal or transdormal suturing of the lower alar cartilage to support and define the tip of the nose or cinch suturing at the alar base for narrowing may be performed. The orbicularis oris muscle with abnormal insertion is dissected and sutured to restore continuity. If there is a vertical deficiency, a small triangular flap or vermilion unilimb Z-plasty may be added. In addition, based on the results of many studies on growth, slow-growing structures, such as the columella and nasal tip, are made slightly larger than normal, whereas the fast-growing structures, such as the philtrum, are made smaller. As an exceptional structure, the median tubercle usually grows rapidly, but this is not the case in patients with bilateral cleft lip after surgery; hence, the median tubercle should be as long as possible [11].

Despite these efforts, unanticipated results emerge after surgery, such as a pointed Cupid’s bow, a flat prolabium, the absence of a philtral dimple, a wide philtrum, a tight upper lip, and the absence of a tubercle. In particular, unlike the philtrum, a slow-growing artificial tubercle forms a secondary deformity gradually due to the inadequate bulk provided by the lateral segment vermilion. Nevertheless, the lateral segment vermilion is used to construct the tubercle because Mulliken’s principle [4], which is the basis of modern surgery, is still followed. (1) Cupid’s bow of the prolabium is rounded; (2) white roll is indistinct and absent in the prolabium; (3) the color of central the vermilion is not the same as that of the lateral lip vermilion; (4) it is difficult to obtain an accurate approximation of vermilion for a good esthetic outcome. The prolabium in the bilateral complete cleft lip is essentially made up of collagenous connective tissue without muscle fibers, and the normal philtrum and Cupid’s bow are also absent. The white roll is not clearly distinguished, and the vermilion color of the prolabium is more red than the vermilion color of the lateral lip. As a result, in conventional techniques, all remaining tissues, except the prolabial flap used to create the philtrum, are discarded. However, efforts are being made to gradually improve the results by making the use of vermilion of the prolabium from part to whole [12–15]. The rationale behind doing this is as follows. The majority of natural lips have gentle curves and do not form an angle, as seen in conventional surgery outcomes. Mulliken et al. described that the white roll prominence is caused by the underlying marginalis orbicularis oris muscles [16]. In contrast to before surgery, the white roll of prolabium placed on the repairing marginalis orbicularis oris shows clearly visible results after surgery. The color of the median vermilion differs from that of the lateral lip vermilion. Proliferated blood vessels, decreased melanin, and increased non-keratinized tissue give a more reddish hue to the vermilion of the prolabium. Histopathologically, it showed marked vascularity with thick-walled and congested blood vessels similar to erectile tissue [14]. This is rather a part that contributes to creating a natural tubercle.

**Fig. 4:**
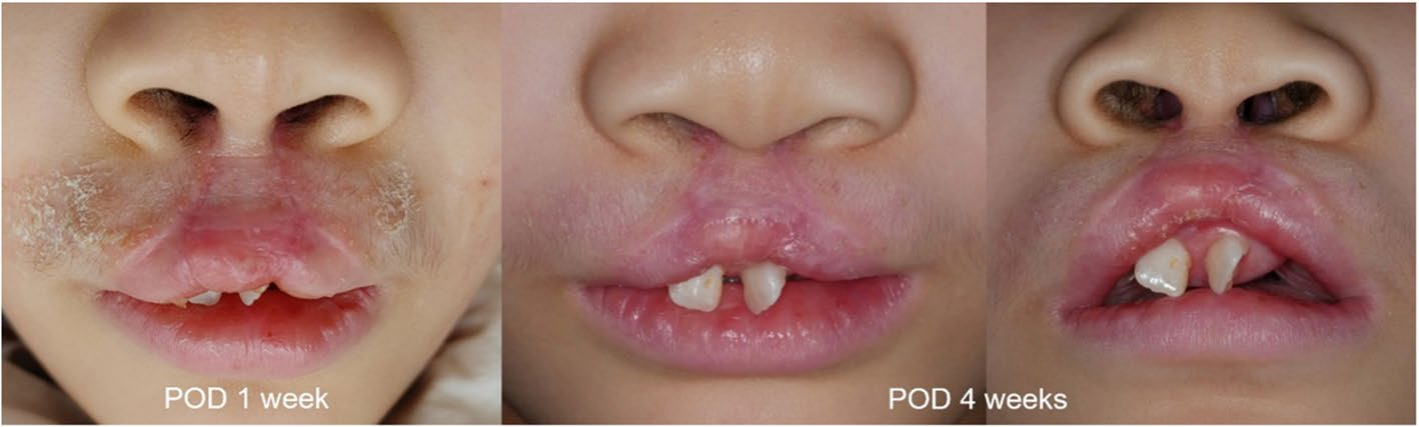
Postoperative photographs

The patient in this case had high skin sensitivity and could not use any dressings, such as compression or steri-strip, after surgery. It is unfortunate that the scar was accentuated by surgical site irritation. However, the prolabial flap was extended to the premaxilla hinge and placed on the orbicularis oris muscle, which had gained continuity, to achieve a white roll, a gently curved vermilion line, and a protruding tubercle and volume. This finding is different from previous surgical results and closer to normal appearance (Fig. 4). Other reports using this method also show better esthetic outcomes and good long-term follow-up results.

In the present case, only the C-flaps that were formed on both sides of the prolabial flap were excised. This approach is consistent with the Mulliken’s report[8] stating that forked flaps are not required to increase the length of the columella. However, they play a role in maintaining blood flow to the prolabial flap as a stable base for manipulation during surgery and in creating a natural U-shaped columellar base by proper trimming at the end. The remaining tissues were elevated as medial vermilion flaps and fully utilized for the oral lining, which contributed to the reduction of tension caused by tissue securing.

The technique of attempting to achieve close to “normal” appearance by preserving the tissue and reflecting the patient’s unique characteristics is similar to Millard's principle, which is “concerned with describing in intricate details a logical way of finding the missing pieces and carefully fitting them into the puzzle so that the final picture is complete, normal, and happy in function and appearance”[17]. The surgical method is applied based on the situation of various cases and each surgeon’s experience. This method, which preserves and utilizes tissue as much as possible and reflects the patient's unique structural characteristics, has a low level of difficulty but a higher esthetic outcome than conventional surgery and shows good long-term results. We believe that in the future, this method will be supported and accepted by more surgeons as the standard.

## Data Availability

Not applicable.
